# A case of systemic juvenile idiopathic arthritis with pulmonary hemosiderosis secondary to recurrent macrophage activation syndrome or a new autoinflammatory syndrome?

**DOI:** 10.1186/1546-0096-13-S1-P54

**Published:** 2015-09-28

**Authors:** K Barut, V Sen, A Adrovic, AB Sinoplu, O Kasapcopur

**Affiliations:** 1 Istanbul University, Cerrahpasa Medical Faculty, Pediatric Rheumatology, Istanbul, Turkey; 2 Dicle University, Medical Faculty, Pediatric Chest Diseases, Diyarbakir, Turkey

## Introduction

Macrophage activation syndrome (MAS), a severe complication of systemic juvenile idiopathic arthritis (JIA) and other inflammatory diseases, represents a one of the most important rheumatological emergencies. Delayed diagnosis could lead to life-threatening complications. Pulmonary hemosiderosis (PH), possible seen at all ages but most commonly among children, usually appears as an idiopathic PH.

## Objective

In this case report of a child with systemic JIA diagnosed at infancy, we aimed to analyze the results of recurrent MAS attacks and to revise the patient's clinical course. The answer being sought in this presentation: does the high ferritin level, being increased secondary to recurrent MAS attacks at a patient with systemic JIA diagnosed at infancy, represent a reason for the PH? Is there a need for further investigations of MAS secondary hemosiderosis?

## Case report

A 13 months old previously healthy infant, admitted to our clinics because of high fever and rash. The fever showed an intermittent course, lasting for two weeks. A physical examination revealed a remarkable hepato-splenomegaly, bilateral wrist and ankle arthritis and a maculopapular, pink coloured rash being prominent especially during high fever. In order to exclude the infectious diseases, viral and bacterial infectious markers were tested and it was found to be negative. A bone marrow biopsy was performed for differential diagnosis of malignant or storage disease: no depot cells or malignant cells were found in the bone marrow.

A persistence of high fever was highly suggestive for systemic JIA and MAS as its secondary complication. The pulse steroid, cyclosporine (CYC) and anakinra were induced in therapy. Diagnosis of systemic JIA secondary MAS was a reason for hospitalization of patient about five times during the one year follow up. During the hospitalization of patient, a ferritin level was found to be as high as 120.990 ng/ml. Due to recurrent MAS attacks, a genetically investigation for familiar hemophagocytic lymphohystiocytosis was performed. The result was negative.

During the last hospitalization (2,5 years old patient), a respiratory difficulties and diffuse infiltrations on chest radiography, accompanied with a high ferritin level and anemia were a reasons to consider a pulmonary hemosiderosis in a differential diagnosis of patient. Thoracic tomography revealed a diffuse fibrous changes and a reticulo-nodular image in lung. Histochemical investigation of broncho-alveolary lavage fluid by the ferrous stain showed iron bearing macrophages. Thereby, a diagnosis was pulmonary hemosiderosis secondary to MAS, one of the most severe complications of systemic JIA.

The pulse steroid therapy was given in order to keep the MAS attacks under control. Prednisone 15 mg/day, methotrexate 5mg/week/oral route, CYC 50mg/day, anakinra 60 mg/day were used as a maintenance treatment.

Since the dyspnea became prominent during the clinical course of the disease, patient was admitted to the intensive care unit. At the time of discharge from the hospital, patient remained dependent on oxygen therapy. Mentioned therapy resulted with patient's good general status with no high fever but the need for permanent oxygen therapy continues.

## Conclusion

Pulmonary hemosiderosis is being divided in a two main grup: idiopathic (primary) and secondary PH. IPH is considered to be more common than secondary, which is thought to be very rare. In the case of MAS, the most important and the most destructive complication of SJIA, ferritin could reach a very high level. Recently conducted a multi-centric study among SJIA secondary MAS cases showed a beginning ferritin level to be high as 8,325 ng/ml (2,048-22,977 ng/ml). None of those cases had a clinical presentation of pulmonary hemosiderosis.

Our patient with SJIA secondary MAS had a ferritin level of 120.990 ng/ml. Prominent dyspnea, reticular image on the chest radiography and anemia were suggestive for diagnosis of pulmonary hemosiderosis. Histochemical investigation of broncho-alveolary lavage fluid by the ferrous stain showing an iron bearing macrophages confirmed the diagnosis of pulmonary hemosiderosis.

For the best of our knowledge, this is the first case of pulmonary hemosiderosis secondary to MAS. In MAS patients with high ferritin level, PH should be considering as a possible complication.

## Consent to publish

Written informated consent for publication of their clinical details was obtained from the patient/parent/guardian/relative of the patient.

